# Comment on Somnin et al. Study of Interactions Between Gadolinium-Based Contrast Agents and Collagen by Taylor Dispersion Analysis and Frontal Analysis Continuous Capillary Electrophoresis. *Pharmaceuticals* 2024, *17*, 1633

**DOI:** 10.3390/ph18081233

**Published:** 2025-08-21

**Authors:** Nicol Guidolin, Federico Maisano, Fabio Tedoldi, Zsolt Baranyai

**Affiliations:** Bracco Imaging Spa, Bracco Research Centre, Via Ribes 5, 10010 Colleretto Giacosa, Italyfederico.maisano@bracco.com (F.M.);

**Keywords:** gadolinium-based contrast agents, collagen, Taylor dispersion analysis, ultrafiltration, HPLC, capillary electrophoresis

With great interest, we read the recent paper published in Pharmaceuticals, titled “Study of Interactions Between Gadolinium-Based Contrast Agents and Collagen by Taylor Dispersion Analysis and Frontal Analysis Continuous Capillary Electrophoresis” [1]. Dealing with the interaction between Gadolinium-Based Contrast Agents (GBCAs) and collagen, it challenged the results that we found and published about 5 years ago [2].

While we appreciate that the interaction between GBCAs and collagen is kept under scrutiny, we read that “*the cellulose-type membrane of the Amicon^®^ device is not appropriate for studying the interaction of GBCA*” and that “*we demonstrated that the GBCA can be retained by the filter (especially with the cellulose filters Amicon^®^ MWCO 10 kDa)*”, where such retention ranged “*from 8.8% to 50%*”. Amicon devices are used everywhere, and their high recovery (i.e., low binding) is highly appreciated. Finding that they can retain up to 50% of small, hydrophilic drugs raises strong concerns.

When we performed experiments in our published studies, we checked our experimental setup, as it is a normal laboratory practice, and did not find any significant aspecific binding by the cellulose membrane of the ultrafiltration device. However, we decided to try to reproduce the test conditions of the current paper to understand how the above unexpected conclusions could be reached.

It was not specified which Amicon device had been used; however, since the Amicon^®^ Ultra-0.5 device is the only one that can withstand the high adopted RCF, it was the only reasonable choice. It was used according to the manufacturer’s instructions [3]. As was indicated in the paper [1], 2.5 and 7 mM solutions of ProHance, Dotarem, and Gadovist at pH = 7.4 in the presence of 10 mM Tris-HCl have been prepared in triplicate. Then, samples were submitted to a 3-min centrifugation at 12,000 rpm and 37 °C in the Amicon^®^ Ultra-0.5 devices. Nearly all the samples went through the membranes and were collected. Their GBCA concentrations (*C*_UFC_) were determined both by HPLC and capillary electrophoresis (CE), then they were compared with the untreated solutions to calculate the % recovery (*C*_UFC_/*C*_0_). The results are reported in Table 1.

Based on the above summarized results (i.e., recovery range 97–102%), one can appreciate that the adopted Amicon devices do not retain significantly any GBCAs, independently of their concentrations, chemical structure, and analytical technique. We may have inadvertently omitted or altered some experimental details compared to the experimental setup adopted in [1], but we cannot explain their grossly different results reported in their Table S1.

We first suspected that the centrifugation conditions might play a role, since, in our paper, we used 3000 rpm for 30 s at 37 °C to extract a minimum volume without perturbing the equilibria in the retained sample. However, after reproducing the published experimental setup, we found the above results, i.e., no retention of GBCAs on the cellulose membrane of the Amicon^®^ Ultra-0.5 device.

Next, we were concerned about the analytical method. While HPLC and CE are universally adopted technologies with optimal analytical performances, Taylor Dispersion Analysis (TDA) is not a well-known technique and is generally not used for quantitative determination of sample concentrations.

We would appreciate it if the authors could address and offer explanations about the issues mentioned above.

There are also a few other concerns about how collagen is treated and fractionated, with procedures that, unlike the original intent of improving reproducibility, may actually complicate the model without improving its similarity with physiological conditions. However, these are a part of scientific discussion and go beyond the intent of this comment, which is centered on the scientific correctness of published results and the apparently unmotivated criticism of our peer-reviewed paper [2].

**Materials and Methods**

For the experiments, commercially available gadobutrol (Gadovist^®^, Gd(BT-DO3A); Bayer Healthcare, Leverkusen, Germany), gadoterate meglumine (Dotarem^®^, Gd(DOTA); Guerbet, Roissy CdG Cedex, France), and gadoteridol (ProHance^®^, Gd(HP-DO3A); Bracco Imaging S.p.a, Milano, Italy) were used.

Capillary electrophoresis (CE): Micellar Electrokinetic Capillary Chromatography (MEKC) was successfully used for the separation and determination of the macrocyclic GBCAs in serum and urine samples [4]. A Hewlett–Packard HP^3D^ (Hewlett-Packard GmbH, Waldbronn, Germany) capillary electrophoresis system was used, and separations were performed using bare fused-silica capillaries of 56 cm × 50 μm i.d. (Agilent, Waldbronn, Germany). Before use, the capillaries were washed with 1.0 M NaOH (15 min), 0.1 M NaOH (30 min), and with the buffer electrolyte (30 min). All buffers had been filtered through a 0.45 μm syringe filter and stored in a refrigerator at + 4 °C. Samples were injected hydrodynamically at the anodic end of the capillary (50 mbar, 20 s). The capillary was preconditioned with the buffer electrolyte (25 mM Na_2_HPO_4_, 70 mM sodium dodecyl sulfate (SDS), pH = 9.1) for 3 min. The separation was performed at 12 °C with the application of 20 kV voltage. After analysis, postconditioning with 0.1 M NaOH (3 min) and buffer (3 min) was applied to remove all possibly adsorbed materials from the capillary. In all measurements, 5 mM DMSO as an internal standard was applied in order to correct the migration time and to normalize the area values of components on the electropherogram. Detection was carried out by on-column DAD measurement. The electropherograms were recorded and processed by a ChemStation (A.06.03 version, Agilent, Waldbronn, Germany). The individual linear regression equations (response–concentration) for each macrocyclic Gd(III)-complex were based on four concentrations. The peak areas were linear (R^2^ > 0.9995) in the concentration range specified in Table 2. Analytical performance of the MEKC method is summarized in Table 2.

HPLC: The experimental setup of the HPLC measurements is summarized in Table 3. The chromatograms were recorded and processed by a ChemStation (35900 A 2.3.54 version, Agilent, Waldbronn, Germany). The individual linear regression equations (response–concentration) for each macrocyclic Gd(III)-complex were based on four concentrations. The peak areas were linear (R^2^ > 0.9995) in the concentration range specified in Table 2. Analytical performance of the HPLC method is summarized in Table 2.

## Figures and Tables

**Table 1:** Recovery (%) of 2.5 and 7.0 mM ProHance, Dotarem, and Gadovist solutions obtained after ultrafiltration with Amicon^®^ Ultra, 0.5 mL MWCO 10 kDa ultrafilter (10 mM TRIS, pH = 7.4, 12,000 rpm, 37 °C, 3 min). Mean values of area after/area before ultrafiltration (*n* = 3). SD values are shown in parentheses.

CE of 2.5 mM GBCAs	ProHance	Dotarem	Gadovist
Recovery (%)	97 (4)	99 (2)	99 (4)
CE of 7.0 mM GBCAs	ProHance	Dotarem	Gadovist
Recovery (%)	98 (4)	100 (1)	102 (1)
HPLC of 2.5 mM GBCAs	ProHance	Dotarem	Gadovist
Recovery (%)	100 (1)	98 (1)	101 (1)
HPLC of 7.0 mM GBCAs	ProHance	Dotarem	Gadovist
Recovery (%)	100 (1)	100 (1)	101 (1)

**Table 2:** Analytical performance of CE and HPLC methods for the determination of GBCAs. Linearity was verified in the concentration range of 1.0–7.0 mM GBCAs.

	CE				HPLC ^a^		
	LOD ^b^ (μM)	LOQ ^c^ (μM)	Slope (mAU·s/M)	λ (nm)	LOD ^b^ (μM)	LOQ ^c^ (μM)	Slope (mAU·s/M)
ProHance	4.2	14.2	(6.64 ± 0.02) × 10^4^	200	0.21	0.69	(4.04 ± 0.03) × 10^5^
Dotarem	1.3	4.5	(2.14 ± 0.04) × 10^5^	195	0.18	0.59	(3.72 ± 0.05) × 10^5^
Gadovist	6.8	22.8	(1.90 ± 0.08) × 10^5^	195	1.4	4.7	(4.00 ± 0.03) × 10^5^

^a^ Detection was performed at 210 nm for the three compounds. ^b^ LOD = 3σ/slope and ^c^ LOQ = 10σ/slope, where σ is the regression standard deviation.

**Table 3:** Experimental setup of the HPLC measurements for ProHance, Dotarem, and Gadovist.

HPLC System	HPLC Equipped with Quaternary Pump, Degasser, Autosampler, and PDA Detector (Agilent 1260 Infinity II, Waldbronn, Germany)		
Stationary phase	Gemini 5 μm C18 110A-150 × 4.6 mm (Phenomenex, Torrance, CA, USA)		
Mobile phase	H_2_O/TFA 0.1%: ACN		
Elution: Isocratic	H_2_O	H_2_O/TFA 0.1%	ACN
	73.5	25	1.5
Flow	1.0 mL/min		
Temperature	25 °C		
Detection	PDA scan wavelenght 190–800 nm		
Injection volume	50 µL		
Sample concentration	1.0–7.0 mM GBCAs		
Stop time	6 min		
Retention time	GdL ≅ 3–4 min		

## Data Availability

The raw data supporting the conclusions of this article will be made available by the authors upon request.

## Abbreviations

The following abbreviations are used in this manuscript:

ACN	acetonitrile
CE	Capillary Electrophoresis
DAD	Diode-Array Detector
DMSO	dimethylsulfoxide
GBCAs	Gadolinium-Based Contrast Agents
HPLC	High-Performance Liquid Chromatography
MEKC	Micellar Electrokinetic Capillary Chromatography
PDA	Photo Diode Array
TDA	Taylor Dispersion Analysis
TFA	trifluoroacetic acid
